# BALB/c Mouse Is a Potential Animal Model System for Studying Acute and Chronic Genotype 4 Hepatitis E Virus Infection

**DOI:** 10.3389/fmicb.2020.01156

**Published:** 2020-06-16

**Authors:** Yunlong Li, Feiyan Long, Chenchen Yang, Xianhui Hao, Jian Wu, Jianwen Situ, Shuangfeng Chen, Zhongyao Qian, Fen Huang, Wenhai Yu

**Affiliations:** ^1^Medical Faculty, Kunming University of Science and Technology, Kunming, China; ^2^State Key Laboratory for Diagnosis and Treatment of Infectious Diseases, National Clinical Research Center for Infectious Diseases, Collaborative Innovation Center for Diagnosis and Treatment of Infectious Diseases, The First Affiliated Hospital, College of Medicine, Zhejiang University, Hangzhou, China; ^3^Institute of Medical Biology, Chinese Academy of Medical Sciences and Peking Union Medical College, Kunming, China

**Keywords:** hepatitis E virus, genotype, BALB/c mice, infectivity, animal model

## Abstract

Hepatitis E virus (HEV) is the main pathogen of hepatitis worldwide. However, its infection biology and pathogenesis remain largely unknown. Suitable small-animal models are required to advance the study of HEV infection. Although an efficient model of genotype 1 (gt1) and gt3 HEV infection has been established in human liver chimeric mice, the infectivity of gt4 HEV infection in mice has not been comprehensively characterized. In this study, immunocompromised BALB/c nude, immunocompetent BALB/c, and C57BL/6 mice were inoculated with either gt3 or gt4 HEV (19 HEV strains, including human, swine, macaque-adapted, and cow HEV strains). Infectivity was identified by viral RNA and antigen detection, inflammation, and histopathological analysis. Then, HEV-infected BALB/c mice were treated with antiviral drugs. Acute HEV infection was established in BALB/c mice inoculated with eight gt4 HEV strains. However, gt3 HEV strains failed to achieve active HEV infection. HEV infection was established in BALB/c nude and regular mice inoculated with gt4 HEV but not in C57BL/6 mice. Gt4 HEV infection resulted in rapid viremia and high titers in feces, sera, and replication sites. HEV infection in mice showed no gender preference. Furthermore, chronic gt4 HEV infection was well imitated in BALB/c mice for 32 weeks and caused liver fibrosis.

**Conclusion:**

BALB/c mice have a great potential for reproducing the process of gt4 HEV infection. The successful establishment of a gt4 HEV small-animal model provides an opportunity to further understand HEV infection biology and zoonotic transmission and develop anti-HEV vaccine.

## Introduction

Hepatitis E virus (HEV) is a viral pathogen that causes acute hepatitis and chronic infection in immunocompromised patients (Kamar et al., 2008; Aggarwal and Jameel, 2011). HEV has one serotype and eight genotypes. Genotype 1 (gt1) and gt2 HEV only infect humans and are mainly prevalent in Asia, Mexico, and Africa. These genotypes are responsible for 20 million infections and 70,000 deaths annually (Rein et al., 2012). Gt3 and gt4 HEV can be transmitted from several animals, including swine, deer, and cow, to humans mainly through the consumption of undercooked meat or milk (Huang et al., 2016a; Rivero-Juarez et al., 2016). HEV infection is thought to be acute and self-limiting, but more chronic infections have been reported recently (Aggarwal and Jameel, 2011; Aggarwal, 2011). Chronic HEV infections, defined as HEV RNA persistently positive in feces or serum for more than 3 months, have been reported in immunocompromised patients, especially in organ-transplant recipients, caused predominately by gt3 and partially by gt4 (Kamar et al., 2008; Abravanel et al., 2014; Perumpail et al., 2015). However, no antiviral medication is available because of the unavailability of suitable animal models. Hence, HEV infection biology and pathogenesis remain largely unknown.

The most important tools for HEV infection and pathogenesis research are small-animal models. However, the development of animal models, particularly the use of small laboratory animals, has not been adequately explored. Although gt1 and gt3 HEV chronic infections of human liver chimeric mice have been established in early 2016 to prove that only humanized liver can be infected with HEV (Allweiss et al., 2016; Sayed et al., 2016; van de Garde et al., 2016), these mice are frail, expensive, and cannot reproduce humanized offspring. BALB/c nude mouse-based gt4 HEV models have been successfully established to simulate immunocompromised patients infected with swine HEV (Huang et al., 2009). The Mongolian gerbil-based HEV model is also susceptive to gt4 swine HEV isolated in China (Li et al., 2009; Soomro et al., 2017). However, the question of whether gt4 HEV that is endemic in China can infect mouse must be resolved.

No HEV-specific treatments are presently available. Ribavirin (RBV), which is primarily used to treat hepatitis C and viral hemorrhagic fevers, is the first-choice therapy for chronic gt3 HEV infection but its use has achieved limited success (Todt et al., 2016b; Barrague et al., 2017). Moreover, resistance and relapse have been widely reported clinically when RBV monotherapy is used in patients chronically infected with HEV (Debing et al., 2014b, 2016; Todt et al., 2016b). As an alternative, interferon-α (IFN-α) (IFN-α1b) has been administered alone or in combination with RBV *in vitro* and *in vivo* (Debing et al., 2014a; Todt et al., 2016a), but severe side effects have also been reported (Behrendt et al., 2014). Sofosbuvir (SOF), an efficient antiviral drug for hepatitis C virus (HCV), is reportedly a potential anti-HEV drug candidate, but some studies have rejected its anti-HEV effect (Wang et al., 2016). Most chronic HEV infection cases are reported in developed countries with endemic gt1 or gt3 HEV, and chronic infection caused by gt4 HEV has rarely been reported. Therefore, whether gt4 HEV infection is sensitive to these antiviral drugs is unknown.

In the present study, BALB/c-based acute and chronic (HEV RNA persistently positive for 32 weeks) gt4 HEV infections were successfully established. We found that immunocompromised and immunocompetent BALB/c or C57BL/6 mice were not susceptible to gt3 HEV. The successful establishment of acute and chronic HEV BALB/c mice models has important implications for exploiting the HEV pathogenesis mechanism and developing drugs against this disease.

## Materials and Methods

### Viruses

Gt3 swine HEV (SAAS-JDY5) isolated from Shanghai was provided by Dr. Zhen Li (Shanghai Academy of Agricultural Sciences). Nineteen Gt4 HEV strains, including swine (KM01), human (LX), chronic-infected rhesus macaque-adapted (macKM01), and cow HEV (milk, 1#–16#) HEV strains, were isolated from nine provinces of China (Table 1). Fecal suspension (10% [w/v]) was centrifuged at 12,000 × *g* at 4°C for 10 min, filtered through 0. 22-μm microfilters, and treated with penicillin and streptomycin for 1 h. Viral genomic titers were determined by quantitative real-time polymerase chain reaction (qRT–PCR), as previously described (Huang et al., 2016a).

**TABLE 1:** HEV strains used in this study.

**StrainGenBank No.**	**Gt**	**Host**	**Location**	**Viral titer (Dose)**	**Infectivity (Rhesus Macaque)**	**Infectivity (regular BALB/c)**	**References**
1#KU974927	Gt4	Cow (milk)	Dali	46,710.12 (300 μl)	N/A	No (po.)	This study
2#KU974928	Gt4	Cow (milk)	Dali	82,612.39 (300 μl)	N/A	No (po.)	This study
3#KU974938	Gt4	Cow (milk)	Dali	148,106.97 (300 μl)	Yes (po.)	Yes (po.)	Huang et al., 2016a
4#KU974946	Gt4	Cow (milk)	Dali	39,687.92 (300 μl)	N/A	Yes (po.)	This study
5#KU974948	Gt4	Cow (milk)	Dali	74,109.99 (300 μl)	N/A	Yes (po.)	This study
6#KU974934	Gt4	Cow (milk)	Dali	325,855.12 (300 μl)	No (po.)	No (po.)	This study
7#KU974935	Gt4	Cow (milk)	Honghe	348,742.35 (300 μl)	Yes (po.)	Yes (po.)	Huang et al., 2016a
8#KU974937	Gt4	Cow (milk)	Meishan	363,239.43 (300 μl)	N/A	No (po.)	This study
9#KU974939	Gt4	Cow (milk)	Yinshan	300,364.22 (300 μl)	N/A	No (po.)	This study
10#KU974940	Gt4	Cow (milk)	Inner Mongolia	346,383.15 (300 μl)	N/A	No (po.)	This study
11#KU974941	Gt4	Cow (milk)	Ningxia	341,712.35 (300 μl)	N/A	Yes (po.)	This study
12#KU974942	Gt4	Cow (milk)	Lanzhou	489,672.87 (300 μl)	N/A	No (po.)	This study
13#KU974943	Gt4	Cow (milk)	Hebei	584,190.84 (300 μl)	N/A	No (po.)	This study
14#KU974944	Gt4	Cow (milk)	Hubei	825,855.64 (300 μl)	N/A	No (po.)	This study
15#KU974951	Gt4	Cow (milk)	Shandong	5994,152.95 (300 μl)	N/A	No (po.)	This study
16#KU974950	Gt4	Cow (milk)	Dali	457,536.57 (300 μl)	Yes (po.)	Yes (po.)	Huang et al., 2016a
KM01KJ155502	Gt4	Swine (feces)	Kunming	988,221.31 (po. 300 μl, iv. 100 μl)	Yes (po.) Yes (iv.)	Yes (po.)	Huang et al., 2016a
LXMF567574	Gt4	Human (feces)	Kunming	584,190.84 (100 μl)	N/A	Yes (iv.)	This study
MacHEVmacHEV	Gt4	Macaque (feces)	Kunming	1040,829.7 (po. 300 μl, iv. 100 μl)	Yes (po.) Yes (iv.)	Yes (iv.)	Huang et al., 2016b
JDY5FJ527832	Gt3	Swine	Shanghai	90,891.09 (100 μl)	N/A	No (iv.)	Zhu et al., 2013

*The viral titer of HEV in milk (copies/mL) is defined as the copy number of HEV in 1 mL milk quantified by qRT-PCR; the viral titer of HEV in fecal sample (copies/g) is defined as the copy number of HEV in 10% (W/V) fecal sample suspensions and quantified by qRT-PCR. Gt, genotype; N/A, not tested; po., *per os*; iv., intravenous.*

### Animal and Viral Inoculation

SPF BALB/c nude (females, 6 weeks old, 16–18 g, *n* = 54), BALB/c (females, *n* = 146; males, *n* = 30; 6 weeks old, 18–20 g), and C57BL/6 mice (females, 6 weeks old, 18–20 g, *n* = 54) were purchased from Shanghai Laboratory Animal Center (China) and maintained in a pathogen-free animal facility. The animal protocols were approved by the Animal Care and Use Committee of Kunming University of Science and Technology. Fecal and serum samples were collected for HEV RNA detection by qRT–PCR and anti-HEV IgG and IgM determination by ELISA prior to the conduct of the study, respectively. The protocol for HEV RNA detection by qRT–PCR was described in our previous study (Huang et al., 2016a). Mice negative to anti-HEV antibodies and HEV RNA were used in this study.

BALB/c mice (females, *n* = 20) were separately inoculated with 20 HEV strains (intravenous injection with 100 μl of fecal suspension or gavage with 300 μl of milk each mouse) to screen which strain of gt3 and gt4 HEV is infectious. Feces were collected twice per week for HEV RNA detection.

**FIGURE 1:**
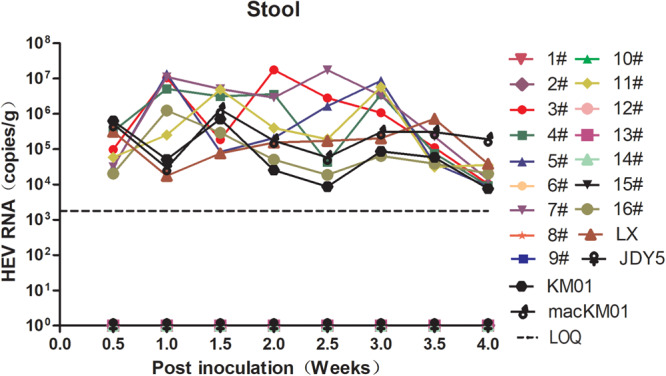
Screening the infectivity of hepatitis E virus (HEV) strains isolated from human, swine, macaque, and cow in BALB/c mice. BALB/c mice were separately inoculated with gt3 or gt4 HEV. Stool samples were collected twice per week and detected by qRT-PCR. The limit of quantification is 2.4 × 10^3^ copies/g.

BALB/c nude, regular BALB/c, and C57BL/6 mice were employed to assess the sensitivity of these strains to gt3 and gt4 HEV. Given that the infectivity of KM01 strain has been confirmed in rhesus macaque (Huang et al., 2016a, b), tree shrew (Yu et al., 2016), and BALB/c mice (Figure 1), it was used to establish the experimental infection of gt4 HEV. Each strain of mice was randomly divided into three groups. As negative control, the mice in group 1 (*n* = 6) were injected with 100 μl of PBS via the tail vein. Those in group 2 (*n* = 24) were injected with 100 μl of stool supernatant of gt4 swine HEV (KM01) via the tail vein. Those in group 3 (*n* = 24) were injected with 100 μl of stool supernatant of gt3 swine HEV (JDY5) by the tail vein. Male BALB/c mice (*n* = 30) were used to determine the sex difference in gt4 HEV infection.

The stool supernatant of chronic-infected rhesus macaque (HEV RNA^+^ in feces lasted more than 670 days, labeled as macKM01), which also caused chronic infection in another rhesus macaque (see details in our previous study; Huang et al., 2016b), was used as the inoculum to establish chronic HEV infection mouse model. BALB/c mice (females, *n* = 36) were injected with 100 μl of the stool supernatant macKM01 through the tail vein.

After viral inoculation, the mice were housed individually. Stool samples were collected twice every week to test the infectivity of HEV in mice, and blood samples were collected weekly. Six mice in each group were euthanized and necropsied at 7, 14, 21, or 28 dpi. For chronic HEV infection, the mice were necropsied at 14 dpi or 32 weeks post-inoculation (wpi, end of the experiment). The liver, spleen, kidneys, intestines, uterus, and brain were collected for HEV RNA detection by using reverse-transcription nested PCR (RT-nPCR) and qRT-PCR. HEV antigens were analyzed at 14 dpi by immunohistochemistry (IHC) and indirect immunofluorescence assay (IFA). Histopathological analyses were performed at 28 dpi for acute HEV infection or 32 wpi for chronic HEV infection.

### HEV Treatment

RBV, IFN-α (IFN-α1b), and SOF were applied in gt4 HEV-infected BALB/c mice to evaluate the effects of antiviral compounds on HEV infection. HEV-infected BALB/c mice (*n* = 36) were randomly divided into six groups according to viral titer in the stool at 4 dpi. The mice in group 1 (*n* = 6) were injected with PBS (negative control). The mice in group 2 were treated with RBV (50 mg/kg/day, orally) for one week. The mice in group 3 were given IFN-α1b (30 μg/kg, intramuscular injection). The mice in group 4 were treated with SOF (400 μg/day/50 kg, orally). The mice in group 5 were administered with a combination of SOF and RBV. The mice in group 6 were treated with a combination of RBV and IFN-α. Stool samples were collected twice every week, and blood samples were collected weekly.

### Virus Detection and Gene Quantification

Total RNA was extracted from the stool (10% suspension [W/V]), blood, serum, and tissues by using Trizol (Invitrogen, United States). Reverse transcription was performed using a reverse transcriptase kit (AMV, Takara, Japan) with HEV-specific negative and positive strand primers or random primer. Nested PCR was conducted according to previous studies (Nanda et al., 1994; Huang et al., 2002, 2016a). Negative control was included to exclude PCR contamination. HEV viral titer was quantified using SYBR Green-based qRT-PCR following our previous study (Huang et al., 2016a).

The relative gene expression of IFN-I, including IFN-α and IFN-β, was quantified with the specific primers as described in a previous study (Xu et al., 2017). GAPDH was used as the housekeeping control gene. qRT-PCR was performed using an ABI PRISM 7300 Real-Time PCR System. Relative gene expression was determined using the formula 2^–(ΔCt of gene–ΔCt of GAPDH)^, where Ct is the threshold cycle.

### Histopathology, IHC, and Indirect Immunofluorescence Analysis

Tissue biopsies were fixed in 10% neutral-buffered formalin and embedded in paraffin. Specimens were cut into 3- to 4-μm serial sections. Standard hematoxylin and eosin or Masson staining was performed, and the tissues were examined under a microscope.

For IHC and indirect IFA analysis, tissues were deparaffinized, hydrated, heated in a water bath for antigen retrieval, and then blocked with the addition of 3% hydrogen peroxide for 15 min. Tissue sections were incubated for 2 h at 37°C with primary antibodies (HEV ORF2, Merck Millipore, Germany; CD45, Abcam, United States; F4/80, Abcam, United States), washed with PBS, and then incubated with HRP-, FITC-, or TRITC-labeled secondary antibody, as described in our previous study (Huang et al., 2018).

### Detection of Anti-HEV IgG and IgM Antibodies

HEV IgG and IgM antibodies were determined using a commercial ELISA kit (Shanghai Kehua Bio-engineering, KHB, China) on the basis of recombinant HEV fusion proteins according to the manufacturer’s instructions, except that the secondary antibodies were replaced with HRP-conjugated antimouse IgM or IgG antibodies (Kirkegaard & Perry Laboratories, KPL, United States).

### Serum Liver Chemistry Profile

Alanine aminotransferase (ALT), aspartate aminotransferase (AST), and total bilirubin (T-Bil) activities in serum were measured using an automated biochemistry analyzer (Mindray BS-200, China).

### Statistical Analysis

Data were presented as mean ± standard deviation. GraphPad Prism software was used for statistical analysis and to determine *p*-values. Student’s *t*-test or χ^2^ analysis was used to determine the significance of differences between two or more groups, in which a 0.05 level of probability (*p* < 0.05) was considered statistically significant.

## Results

### Genotype 4 HEV Is Infectious in BALB/c Mice but Not in Every Strain

In a previous study, we reported that BALB/c nude mice are sensitive to gt4 swine HEV infection isolated from Shanghai, China. Moreover, Mongolian gerbils, also a rodent, are susceptible to gt4 swine HEV isolated from Yunnan, China (Li et al., 2009; Soomro et al., 2017). The matter of whether all gt4 HEV strains are infectious in mouse animal models is unsettled. We screened 20 HEV strains, including 1 gt4 human, 1 gt3 swine, 1 gt4 swine, 1 gt4 macaque, and 16 gt4 cow HEV strains, isolated from 11 cities in nine provinces of China. Only nine of them were infectious in BALB/c mice with detectable HEV RNA shedding in feces (Figure 1 and Table 1). However, the question why BALB/c mice were not susceptible to the other 10 gt4 HEV strains remains inexplicable, despite the fact that these strains were isolated from the same village with higher viral titers and stored in the same environment (Table 1).

Given that not every HEV strain was infectious in BALB/c mice, the question of whether all rodent species are sensitive to infectious gt4 HEV is unresolved. Therefore, we further screened the infectivity of gt4 HEV in common rodent models, including BALB/c nude, BALB/c regular, and C57BL/6 mice.

### Immunocompromised BALB/c Nude Mice Are Sensitive to gt4 HEV but Not Susceptible to gt3 HEV

**FIGURE 2:**
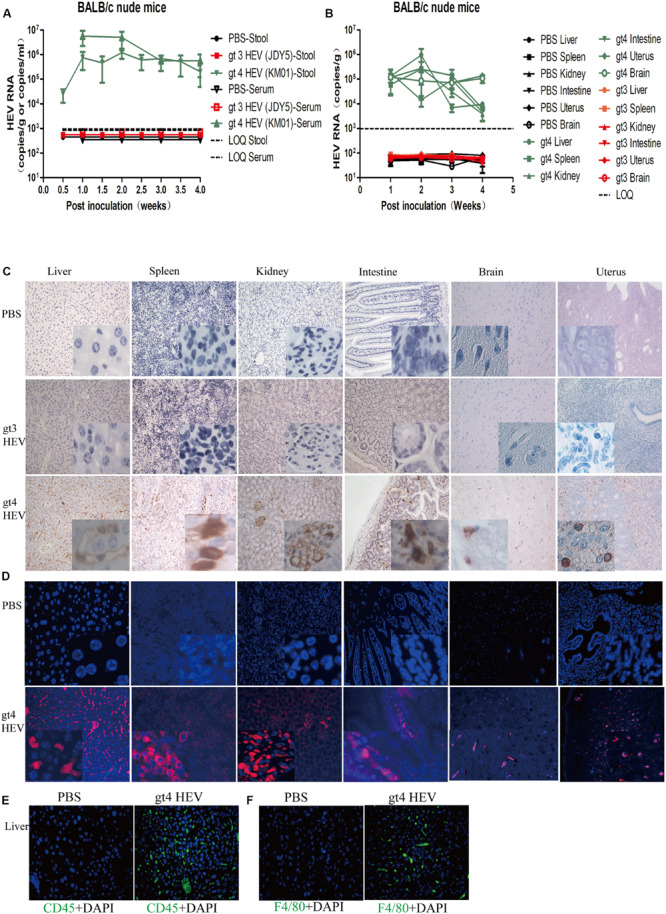
Profiles of gt3 and gt4 hepatitis E virus (HEV) infection in BALB/c nude mice. HEV RNA was detected in the stool and serum **(A)** and tissues **(B)** of BALB/c nude mice inoculated with PBS, gt3 HEV, or gt4 HEV. LOQ, limit of quantification. HEV antigen was detected in the liver, spleen, kidneys, intestines, brain, and uterus of BALB/c nude mice by immunohistochemical method (×200; **C**) and immunofluorescence analysis (×200; **D**). The nuclei were stained with DAPI (blue). Immunofluorescent staining for leukocyte (CD45^+^, green, **E**) or macrophages (F4/80^+^, green, **F**) was performed in the liver of uninfected (PBS) or gt4 HEV-infected BALB/c nude mice, ×400. LOQ is 1.0 × 10^3^ copies/g or copies/ml.

In the present study, nude mice were intravenously inoculated with gt4 HEV (KM01 strain), whose infectivity has been confirmed in rhesus macaque (Huang et al., 2016a, b), tree shrew (Yu et al., 2016), and BALB/c mouse (Figure 1). Similar to rhesus macaque and tree shrew, all nude mice inoculated with KM01 strain were infected with HEV, with shedding of viruses in stool samples starting from 3 to 7 dpi and ending at 4 wpi (Figure 2A). Viremia was detected from 1 to 4 wpi (Figure 2A). Negative strand of HEV RNA was detected in all the liver of HEV-infected nude mice at 7 dpi by RT-nPCR, which indicated the replication of HEV. Positive strands of HEV RNA were detected and quantified by qRT–PCR (Figure 2B). In the other extrahepatic sites, spleen, kidneys, intestines, uterus, and brain, HEV RNA was also detectable from 1 to 3 wpi and sharply decreased at 4 wpi (Figure 2B). However, HEV RNA was undetectable in the stool, serum, or tissue samples of nude mice inoculated with PBS or gt3 HEV during the entire experiment (Figures 2A,B).

IHC and indirect IFA clearly showed HEV antigens in these HEV replication sites in gt4 HEV-infected nude mice at 14 dpi (Figures 2C,D). Positive stains were obviously observed in the liver, spleen, kidneys (glomerulus), intestines, uterus, and brain (cone cells) of nude mice inoculated with gt4 HEV (KM01), whereas no stains were perceived in nude mice inoculated with PBS or gt3 HEV (JDY5) (Figures 2C,D). The presence of HEV RNA and HEV antigen in HEV replication sites firmly confirmed that gt4 HEV can replicate in immunocompromised BALB/c nude mice.

Acute HEV infection usually causes inflammation. Analysis of inflammation strongly indicated that gt4 HEV infection caused acute inflammatory response with increased CD45^+^ leukocyte and F4/80^+^ macrophage in the liver of HEV-infected nude mice (Figures 2E,F). Histopathological analysis also revealed mild inflammation in the liver, spleen, and kidneys of gt4 HEV-infected nude mice (Supplementary Figure 1A). No substantial damage was observed in the intestines, brain, and uterus of gt4 HEV-infected nude mice (Supplementary Figure 1A). No significant change in ALT and AST activities was determined in gt4 HEV-infected nude mice compared with uninfected control mice (PBS group) or mice inoculated with gt3 HEV (gt3 HEV JDY5 group) (Supplementary Figures 1B,C). Anti-HEV IgM and IgG antibodies in gt4 HEV-infected nude mice were negative (Supplementary Figures 1D,E). Shedding of HEV progeny viruses in the feces, detectable HEV RNA and HEV antigen in HEV replication sites, and mild acute inflammatory response stably confirmed that gt4 HEV is capable of successfully infecting immunocompromised BALB/c nude mice.

Gt3, which is another zoonotically transmitted HEV, cannot infect C57BL mice (Todt et al., 2016a). Its infection results in low HEV RNA level in serum and undetectable ORF3 protein in liver of humanized UPA/SCID/beige mice (Allweiss et al., 2016). Similarly, BALB/c nude mice inoculated with gt3 HEV (JDY5) were negative for HEV RNA in all stool and serum samples (Figures 2A,B). Moreover, HEV RNA and HEV antigen were not detectable in the liver, spleen, kidneys, intestines, brain, or uterus (Figures 2B,D). No significant damage was observed in the tissues of nude mice inoculated with gt3 HEV compared with mice treated with PBS (Supplementary Figure 1A). ALT and AST activities exhibited no obvious change (Supplementary Figures 1B,C). Anti-HEV IgG and IgM antibodies were negative (Supplementary Figures 1D,E). These results demonstrated that gt3 HEV cannot infect BALB/c nude mice. Gt3 HEV infection in humans is asymptomatic. The mild characteristics of gt3 HEV may be responsible for the unresponsiveness in mice, even in immunocompromised mice.

### Regular BALB/c Mice Are Also Sensitive to gt4 HEV

Gt4 HEV successfully replicates in immunocompromised BALB/c nude mice and causes classical acute hepatitis E infection. However, the answer to whether immunocompetent regular BALB/c mice, which have a normal immune system, can be infected with gt4 HEV remains elusive. In this study, gt4 HEV was inoculated into immunocompetent BALB/c regular mice. Both female and male BALB/c mice were used to determine whether sex difference affects the infectivity of HEV.

**FIGURE 3:**
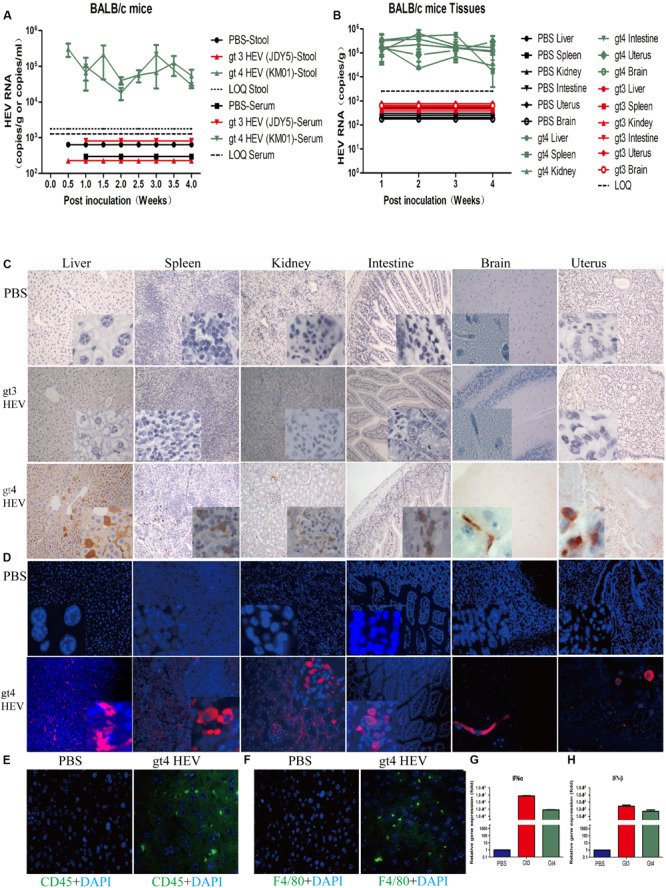
Profiles of gt3 and gt4 hepatitis E virus (HEV) infection in BALB/c mice. HEV RNA was detected in the stool and serum **(A)** and tissues **(B)** of BALB/c mice inoculated with PBS, gt3 HEV, or gt4 HEV. LOQ, limit of quantification. HEV antigen was detected in the liver, spleen, kidneys, intestines, brain, and uterus of BALB/c mice by immunohistochemical method (×200; **C**) and immunofluorescence analysis (×200; **D**). The nuclei were stained with DAPI (blue). Immunofluorescent staining for leukocyte (CD45^+^, green, **E**) or macrophages (F4/80^+^, green, **F**) was performed in the liver of uninfected (PBS) or gt4 HEV-infected BALB/c mice, ×400. The gene expression of IFN-α **(G)** and IFN-β **(H)** was quantified by qRT-PCR in the blood of BALB/c mice inoculated with gt3 HEV or gt4 HEV. LOQ of serum is 1.2 × 10^3^ copies/ml, LOQ of stool is 1.7 × 10^3^ copies/g, and LOQ of tissues is 2.5 × 10^3^ copies/g.

Gt4 HEV was successfully replicated in BALB/c mice. The progeny viruses were shed in the feces of BALB/c mice from 3–7 dpi to 25 dpi. Viremia lasted from 1 wpi to 4 wpi in BALB/c mice inoculated with gt4 HEV (Figure 3A). Negative and positive strands were detected in the liver, spleen, kidneys, intestines, brain, and uterus (Figure 3B). However, no significant difference was observed in the viral titer in the feces, blood, and liver between female and male BALB/c mice, except that the titer was higher in females than in males at 4 wpi (Supplementary Figures 2A–C). Therefore, female BALB/c mice were used in the subsequent experiments. IHC clearly showed HEV antigens in the liver, spleen, kidneys, intestines, brain, and uterus of regular BALB/c mice inoculated with stool-derived gt4 HEV, whereas no positive stains were observed in BALB/c mice inoculated with PBS or stool-derived gt3 HEV (Figure 3C). Indirect IFA clearly revealed positive fluorescent particles of HEV (red) in the liver, spleen, kidneys, intestines, brain, and uterus of BALB/c mice inoculated with gt4 HEV. However, no signal was detected in BALB/c mice inoculated with PBS or gt3 HEV (Figure 3D).

Similar to gt4 HEV-infected BALB/c nude mice, acute inflammatory response was observed in regular BALB/c mice inoculated with gt4 HEV with increased CD45^+^ leukocyte and F4/80^+^ macrophage in the liver of HEV-infected regular BALB/c mice (Figures 3E,F). Mild histopathological changes were detected in the liver (enlarged, focal hepatocellular necrosis), spleen (infiltrating lymphocytes and macrophages), kidneys (congestion), and intestines (infiltrating lymphocytes) of BALB/c mice infected with gt4 HEV. However, no damage was observed in BALB/c mice inoculated with PBS or gt3 HEV (Supplementary Figure 2D).

To evaluate the difference in immune responses against gt3 and gt4 HEV infection, the gene expression of type I interferon (IFN-I, IFN-α and IFN-β), the most important antiviral factors of host, were determined. Interestingly, the expression of IFN-α in gt4 HEV-infected mice was 93.77-fold lower than that in gt3 HEV-inoculated mice (Figure 3G). Similarly, the IFN-β expression in gt4 HEV-infected mice decreased 5.23-fold than that in gt3 HEV-inoculated mice (Figure 3H). The substantial reducing of host antiviral responses caused by gt4 HEV may contribute to the efficient infection in BALB/c mice.

Similar to the clinical features of acute HEV infection, gt4 HEV-infected BALB/c mice showed significantly increased ALT and AST activities at 1–4 wpi and recovered subsequently. However, ALT and AST activities showed no significant change in BALB/c mice inoculated with PBS or gt3 HEV (Supplementary Figures 2E,F). However, adaptive immunity was not evoked in gt4 HEV-infected BALB/c mice (Supplementary Figures 2G,H). Results strongly demonstrated that BALB/c mice are susceptible to stool-derived gt4 HEV but not to gt3 HEV.

### C57BL/6 Mice Are Insensitive to gt4 HEV

**FIGURE 4:**
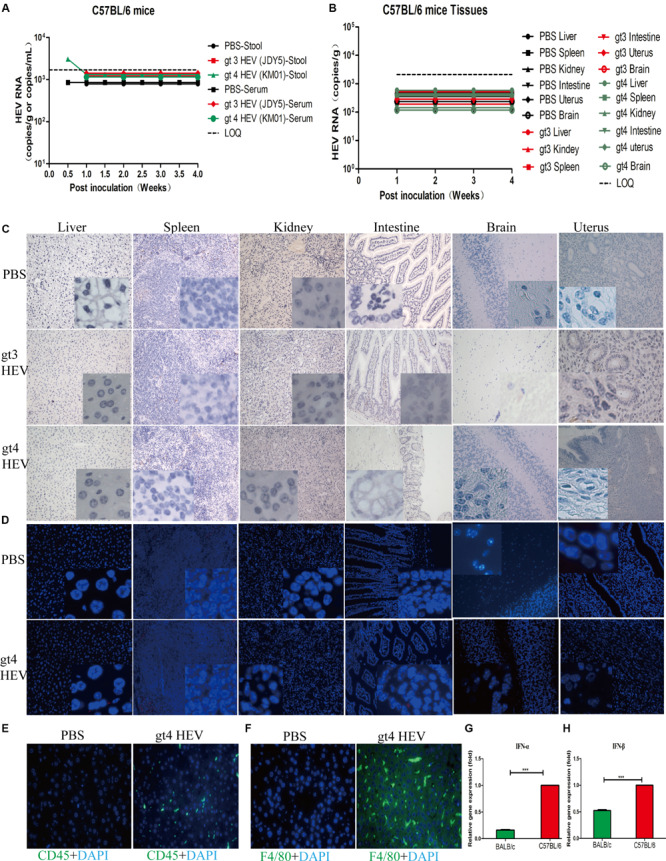
Profiles of gt3 and gt4 hepatitis E virus (HEV) infection in C57BL/6 mice. HEV RNA was detected in the stool and serum **(A)** and tissues **(B)** of C57BL/6 mice inoculated with PBS, gt3 HEV, or gt4 HEV. LOQ, limit of quantification. HEV antigen was detected in the liver, spleen, kidneys, intestines, brain, and uterus of C57BL/6 mice via immunohistochemical method (×200; **C**) and immunofluorescence analysis (×200; **D**). The nuclei were stained with DAPI (blue). Immunofluorescent staining for leukocyte (CD45^+^, green, **E**) or macrophages (F4/80^+^, green, **F**) was performed in the liver of uninfected (PBS) or gt4 HEV-infected C57BL/6 mice, ×400. The gene expression of IFN-α **(G)** and IFN-β **(H)** was quantified by qRT-PCR in the blood of gt4 HEV-infected/inoculated BALB/c mice or C57BL/6 mice at 7 dpi. LOQ of stool and serum is 1.7 × 10^3^ copies/g or copies/ml, and LOQ of tissues is 2.1 × 10^3^ copies/g.

The susceptibility of C57BL/6 mice to HEV infection has been refuted in a previous study in which the animals were inoculated with stool suspensions of gt1, gt3, and gt4 HEV isolated from Japan (Li et al., 2008). In the present study, we further identified the insensitivity of C57BL/6 mice to stool-derived gt4 HEV isolated from China. No HEV RNA was detectable in the feces, blood, or tissues (Figures 4A,B), and no HEV antigen was observed in the replication sites of HEV in spite of gt3 or gt4 HEV inoculation (Figures 4C,D). However, severe immune responses were observed in the enlarged spleen with obvious necrotic foci in C57BL/6 mice inoculated with stool-derived gt4 HEV (Supplementary Figure 3A). This result may have been caused by the overactive immune system in C57BL/6 mice against viral infection than BALB/c mice. Compared with BALB/c mice, more serious immune responses were evoked in C57BL/6 mice with higher increased CD45^+^ leukocyte and F4/80^+^ macrophage in the liver (Figures 4E,F). Meanwhile, the expression of IFN-α and IFN-β in C57BL/6 mice were significantly exacerbated than in BALB/c mice at 7 dpi (Figures 4G,H).

Moderate histopathological changes were observed in the liver with increased lymphocytes and macrophages, but focal necrosis was found in the spleen of C57BL/6 mice inoculated with gt4 HEV (Supplementary Figure 3B). ALT and AST activities showed a temporal increase in the 1st week of gt3 or gt4 HEV inoculation possibly because of a rejection reaction against foreign substances, but the activities recovered subsequently (Supplementary Figures 3C,D). The powerful innate antiviral immunity frustrated HEV infection in C57BL/6 mice; therefore, humoral response against HEV was not provoked (Supplementary Figures 3E,F). Our results confirmed again that C57BL/6 mice are insensitive to gt3 and gt4 HEV infection.

### Successful Establishment of Chronic Infection of gt4 HEV in BALB/c Mice

Cases of chronic HEV infection are recently increasing. However, the progress of HEV chronicity is unclear. Human liver chimeric mice have been recently used as a model for studying gt3 chronic HEV infections (Allweiss et al., 2016). They are useful animal models for the *in vivo* study of HEV infection and drug evaluation. However, these immune-deficient humanized mice, which lack B and T lymphocytes and NK cells, are fragile and expensive.

**FIGURE 5:**
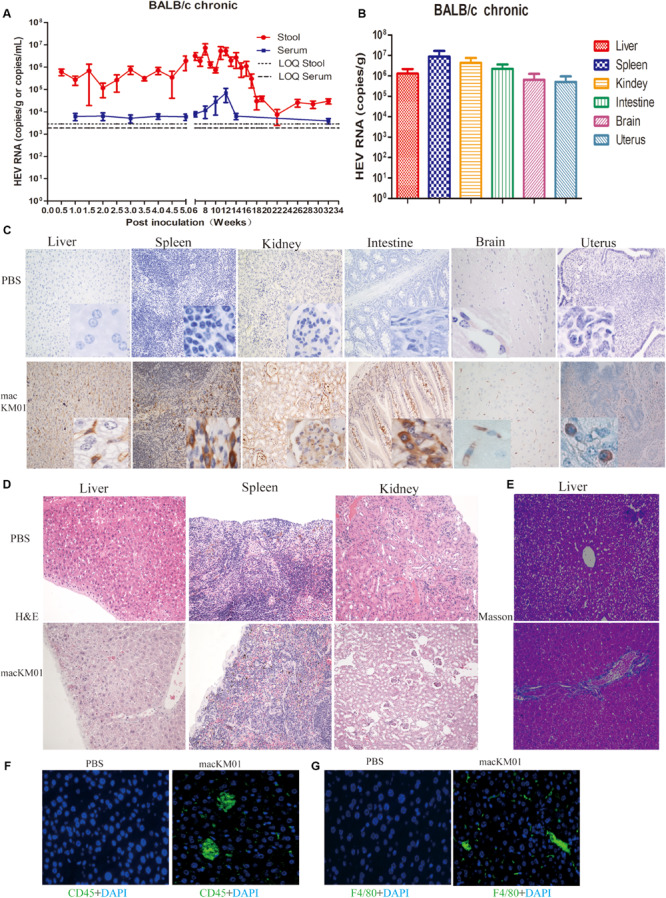
Profiles of gt4 HEV-persistent infection in BALB/c mice. HEV RNA was detected in the stool and serum **(A)** and tissues **(B)** of BALB/c mice inoculated with the stool supernatant from a chronic infected rhesus macaque (macKM01). LOQ, limit of quantification. HEV antigen was detected in the liver, spleen, kidneys, intestines, brain, and uterus of BALB/c mice by immunohistochemical method (×200; **C**) in BALB/c mice inoculated with macKM01, ×200. Histopathological analysis was performed in the liver, spleen, and kidneys of HEV persistent infected BALB/c mice (H&E), ×200 **(D)**. Liver fibrosis was evaluated by Masson staining **(E)**, ×200. Immunofluorescent staining for leukocyte (CD45^+^, green, **F**) or macrophages (F4/80^+^, green, **G**) was performed in the liver of uninfected (PBS) or macKM01-infected BALB/c mice, ×400. LOQ of stool is 2.8 × 10^3^ copies/g, LOQ of serum is 1.8 × 10^3^ copies/ml, and LOQ of tissues is 2.0 × 10^3^ copies/g.

Given that we had successfully established acute gt4 HEV infection in both immunocompromised and immunocompetent BALB/c mice, we evaluated the possibility of chronic HEV infection in BALB/c mouse model by using a rhesus macaque-adapted gt4 chronic-mutated HEV strain (macKM01, stool sample from an HEV-persistent-infected rhesus macaque; for details, see our previous study; Yu et al., 2016). The infectivity of macKM01 strain has been further identified in another rhesus macaque (Yu et al., 2016). HEV RNA was persistently positive, lasting for 32 weeks (end of the experiment) in the stool and serum samples of regular BALB/c mice (Figure 5A). HEV was efficiently replicated in the first 14 weeks, and viral titers in stool samples were persistently increased to 3.0 × 10^7^ copies/ml at 8–12 wpi and slowly decreased at 22 wpi but were still detectable at 32 wpi (end of the experiment, Figure 5A). Although viral titers were relatively lower in the blood than in the stool samples, HEV RNA was still persistently detectable after 32 weeks (Figure 5A). HEV RNA was detected in the liver, spleen, kidneys, intestines, brain, and uterus of BALB/c mice at 32 wpi (Figure 5B). HEV antigens were clearly observed in these HEV replication sites, including the liver, spleen, kidneys, intestines, brain, and uterus, at 32 wpi (Figure 5C). BALB/c mice inoculated with chronic gt4 HEV strain (macHEV) exhibited severe histopathological damages in the liver (infiltrating lymphocytes), enlarged spleen (infiltrating lymphocytes and macrophages), disturbed renal tubular function, and glomerulitis (Figure 5D). Expanded portal tract and proliferative fibrosis were observed in the liver of BALB/c mice infected with the stool of chronic-infected rhesus macaque (macHEV) at 32 wpi (Figure 5E) by using Masson staining. Severe immune responses were evoked with obviously increased CD45^+^ leukocyte and F4/80^+^ macrophage in the liver of BALB/c mice inoculated with chronic gt4 HEV (macHEV) (Figures 5F,G).

### Anti-HEV Drug Treatment

**FIGURE 6:**
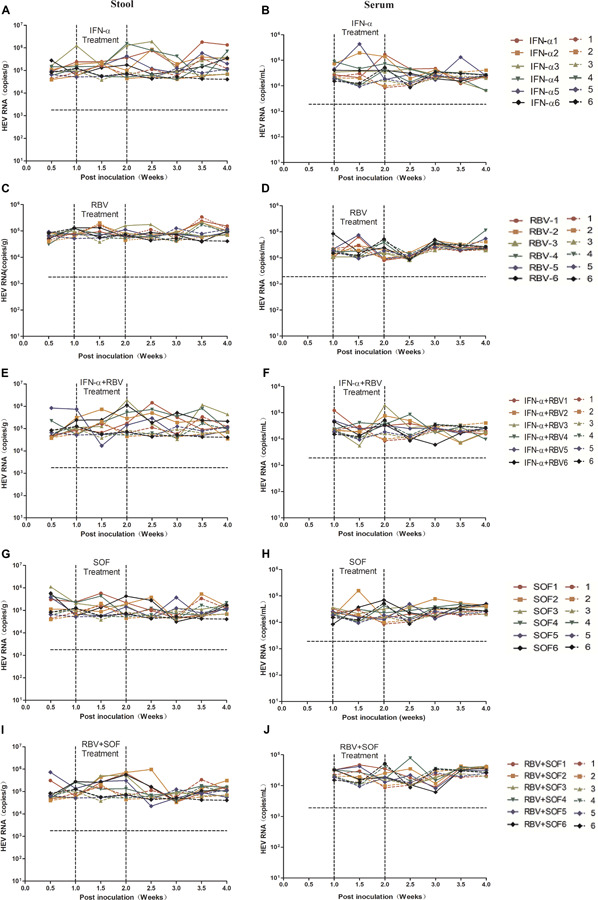
Anti-HEV drug treatment of gt4 HEV-infected BALB/c mice. HEV RNA copy number was quantified by qRT-PCR in the stool **(A,C,E,G,I)** and serum **(B,D,F,H,J)** in gt4 HEV-infected BALB/c mice treated with anti-HEV drugs, including IFN-α, RBV, and SOF monotherapy or combination. LOQ of stool is 1.7 × 10^3^ copies/g, LOQ of serum is 1.8 × 10^3^ copies/ml.

Given that gt4 HEV infection in BALB/c mice had been successfully established, we used this small-animal model to evaluate the anti-HEV effects of RBV, IFN-α, or SOF. The viruses were not completely cleared in both stool and serum samples of gt4 HEV-infected mice with either monotherapy of RBV, IFN-α, SOF, or combination therapy for 1 week (Figure 6). Although RBV monotherapy successfully treated some cases of gt3 HEV chronic infection (Kamar et al., 2014; Allweiss et al., 2016), it failed to treat gt4 HEV (Figure 6). Unsuccessful results were also reported in a patient with gt4 HEV chronic infection who underwent liver transplant and treated with the aforementioned drugs (Wu et al., 2017) possibly because of the mutation of G1634R in the polymerase region of ORF1, an identified single-nucleotide variation resistant to RBV treatment. Although gt1 and gt3 HEV infections were cleared from the liver and feces of humanized mice after pegIFNα injection, relapse was identified in half of treated animals (van de Garde et al., 2017). It was notable; viral replication was unaffected in gt4 HEV-infected mice when treated with either RBV/IFN-α monotherapy or combination therapy (Figures 6A–F). SOF is an efficient antiviral drug for HCV and was once recognized as a potential anti-HEV drug candidate (Thi et al., 2015). However, gt4 HEV-infected mice were insensitive to SOF monotherapy or in combination with RBV (Figures 6G–J), consistent with the clinical failure in patients with gt3 HEV infection (HEV/HCV co-infected) who underwent liver transplant (Wang and Pan, 2016; Donnelly et al., 2017). Long-term anti-HEV treatment with RBV, IFN-α, or SOF reportedly on host immune systems leads to severe side effects, such as elevated ALT, AST, or T-Bil. However, short-term treatment in HEV-infected mice did not aggravate liver damages (Supplementary Figure 4).

## Discussion

Hepatitis E virus is responsible for approximately 20 million infections annually worldwide. However, animal models for *in vivo* studies of HEV infection and pathogenesis are limited. Therefore, developing effective experimental models for understanding HEV biology, pathogenesis, and anti-HEV drug development is important. Although several cell culture systems of HEV have been established (Okamoto, 2011; Meister et al., 2019), small-animal models are urgently needed for studying HEV pathogenicity and antiviral drug development.

Non-human primates were used in the early years of HEV research (Panda et al., 2000; Aggarwal et al., 2001). However, they are no longer employed because of ethical considerations. Swine is unsuitable as an experimental model because of its large body. By contrast, mice are the most commonly used animals for research purposes. As a new model, human liver chimeric mice have been successfully established for gt1 and gt3 HEV infection studies (Allweiss et al., 2016; van de Garde et al., 2016). However, gt4 HEV is different from gt1 or gt3 HEV; the clinical presentations are more severe in gt4 HEV than in gt3 HEV (Jeblaoui et al., 2013). Furthermore, human liver chimeric mice are expensive, fragile, and infertile. Thus, establishing a regular or normal mice-based gt4 HEV model is important for studying HEV infection and pathogenesis.

We have successfully established a gt4 swine HEV (isolated from Shanghai, China) mouse model by using BALB/c nude mice (Huang et al., 2009). The immunodeficient nature of nude mice provides an advantage for investigating HEV infection in immunodeficient patients, such as organ-transplant recipients or patients with HIV infection. In this study, we successfully infected BALB/c nude mice with gt4 stool-derived swine HEV strain (KM01) isolated from Yunnan Province, China (Figure 2 and Supplementary Figure 1). However, most HEV-infected patients are immunocompetent. Thus, regular BALB/c mice, which have a competent immune system, are the most suitable animal models for exploring HEV pathogenesis.

In the present study, only 9 of 20 HEV strains were infectious in BALB/c mice. The diversity of viral genome may have contributed to the infectivity in BALB/c mice because some unique amino acids in ORF2 were found in these strains, resulting in efficient infection (Supplementary Figure 5). However, further experiments, such as metagenomic sequencing to identify potentially critical polymorphisms within these virus genomes and construction of infectious cDNA clones by using reverse genetics to identify these critical mutations, should be conducted. BALB/c mice were successfully infected with gt4 HEV (KM01 strain). HEV RNA was detected in feces starting from 3 to 7 dpi, similar to our gt4 swine HEV strain inoculated in BALB/c nude mice (Huang et al., 2009) but earlier than that inoculated in pigs with gt4 swine RNA transcripts (7 dpi) (Cordoba et al., 2012) and in SD rat inoculated with gt4 swine HEV (Zhu et al., 2013). HEV RNA was detectable in the HEV-replicated sites, including the liver, spleen, kidneys, intestines, brain, and uterus, and this result stably confirmed the extrahepatic replication sites of HEV. HEV replication has recently been identified in the kidneys (Geng et al., 2015; Pischke et al., 2017), brain (Salim et al., 2017; Zhou et al., 2017), and reproductive organs (Soomro et al., 2017; Huang et al., 2018) of patients infected with HEV. HEV RNA and antigens were all detectable in these replication sites and will thus facilitate studies on HEV pathogenesis and tissue tropism.

By contrast, gt3 HEV is dull in mice not only in immunocompromised SCID (Allweiss et al., 2016) or BALB/c nude mice but also in immunocompetent BALB/c or C57BL/6 mice. The capsid protein is the major component of HEV virions to recognize host receptor(s). The distinct differences in capsid protein between gt3 and gt4 HEV may contribute to the susceptibility in BALB/c mice. More gt3 HEV strains should be assessed in BALB/c mice to evaluate the infectivity. In addition, gt3 is prevalent in developed countries, whereas gt4 is mainly reported in Asia and believed to be more pathogenic (Jeblaoui et al., 2013). Consistent with previous reports, C57BL/6 mice were not permissive for both gt3 and gt4 HEV infection. Strong host innate immune system or specific host factor expression may have contributed to the failure of infection.

Chronic HEV infection is increasingly being reported in immunosuppressed patients with HIV infection, hematological malignancy, and organ-transplant recipients. Cirrhosis and liver failure post chronic HEV have been reported (Kamar et al., 2014; Barrague et al., 2017). However, the mechanism of chronic HEV infection remains unclear. Immunosuppression may be the main cause of persistent infection, and viral mutation also cannot be dismissed. We successfully established a macaque-adapted HEV chronic infection mouse model with liver fibrosis and persistent shedding of virus in serum and stool for 32 wpi (8 months). This model will expand our knowledge of HEV chronic infection. However, no robust cell culture system for large-scale propagation of HEV has been established, limiting in-depth research on HEV. We found a macaque-adapted HEV strain that can persistently infect rhesus macaques and BALB/c mice. Establishing a new and promising cell culture system for HEV research is now possible because a stable HEV-infected animal model has been achieved.

Hitherto, no specific treatment for HEV has been approved. Although RBV, which is an off-label drug, is promising because it can inhibit HEV replication by depleting intracellular GTP pool, G1634R mutation is reportedly associated with resistance of RBV therapy in acute and chronic hepatitis E patients (Dalton and Kamar, 2016). Thus, a new specific antiviral therapy for curing chronic HEV infection must be developed. In the present study, RBV, IFN-α, SOF monotherapy, or combination therapy was used to treat gt4 HEV infection in the mouse model. None of these antiviral drugs worked satisfactorily. Short-term, low-dose treatment of antiviral drugs may be responsible for the negativity. Higher compound concentration and longer treatment should be performed in the further study. Thus, the mouse model should be used to develop safe and effective treatment modalities for HEV infection.

## Conclusion

We successfully established a mouse model for acute and chronic gt4 HEV infection in immunocompromised BALB/c nude and immunocompetent BALB/c mice. Although only 50% of natural gt4 HEV strains could establish successful infection in BALB/c mice, BALB/c mice is a potential model system for studying acute and chronic gt4 HEV infection. This system can be used to study the pathogenesis of gt4 HEV and develop anti-HEV drugs.

## Data Availability Statement

All datasets generated for this study are included in the article/Supplementary Material.

## Ethics Statement

The animal study was reviewed and approved by the Animal Care and Use Committee of Kunming University of Science and Technology.

## Author Contributions

YL, FL, CY, and XH performed the experiment. JW, JS, SC, and ZQ contributed to keep the animals and samples collection. FH and WY designed and wrote the manuscript. All authors contributed to the analysis and interpretation of data.

## Conflict of Interest

The authors declare that the research was conducted in the absence of any commercial or financial relationships that could be construed as a potential conflict of interest.
