# Supplementary material for: BALB/c Mouse Is a Potential Animal Model System for Studying Acute and Chronic Genotype 4 Hepatitis E Virus Infection
Source: Front Microbiol. 2020 Jun 16;11:1156. doi: 10.3389/fmicb.2020.01156 (PMC7308725; doi:10.3389/fmicb.2020.01156)

Supplementary file to:

BALB/c mouse is a potential animal model system for studying acute and chronic genotype 4 hepatitis E virus infection

Yunlong Li[1,#], Feiyan Long[1,#], Chenchen Yang[1,#], Xianhui Hao[1,#], Jian Wu[2], Jianwen Situ[1], Shuangfeng Chen[1], Zhongyao Qian[1], Fen Huang[1,*], Wenhai Yu[3,*]

Supplementary Fig.1. Profile of HEV infection in BALB/c nude mice.

Histopathological damage in BALB/c nude mice (H&E) (A). The activities of ALT (B) and AST (C) in BALB/c nude mice. The anti-HEV IgM (D) and IgG (E) antibodies in BALB/c nude mice inoculated with PBS, gt3 HEV, or gt4 HEV.

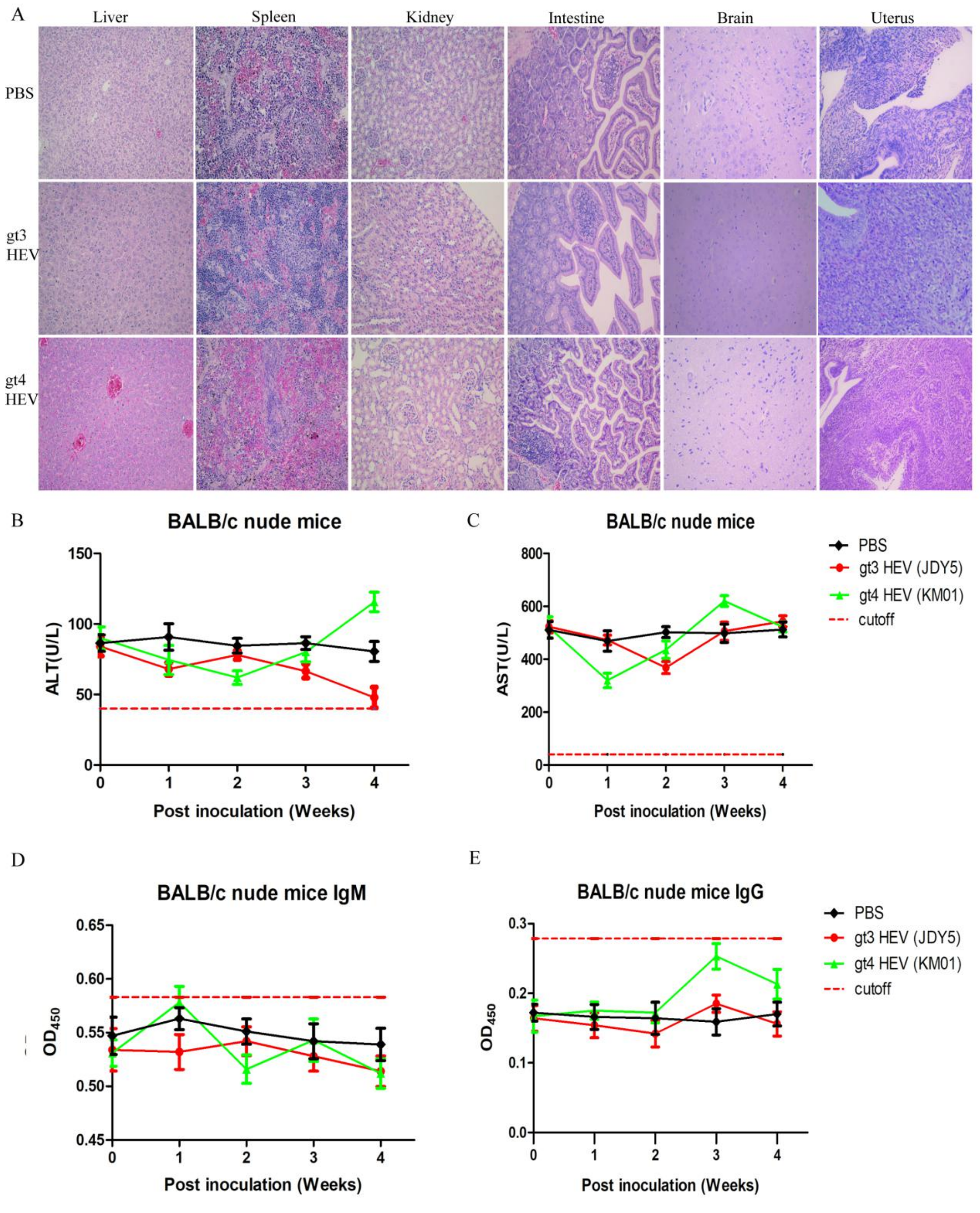

Supplementary Fig.2. Profile of HEV infection in BALB/c mice.

HEV RNA detection in the stool (A), blood (B) and livers (C) of regular BALB/c female or male mice inoculated with gt4 HEV. Histopathological damage in BALB/c mice (H&E) (D). The activities of ALT (E) and AST (F) in BALB/c mice. The anti-HEV IgM (G) and IgG (H) antibodies in BALB/c mice inoculated with PBS, gt3 HEV, or gt4 HEV.

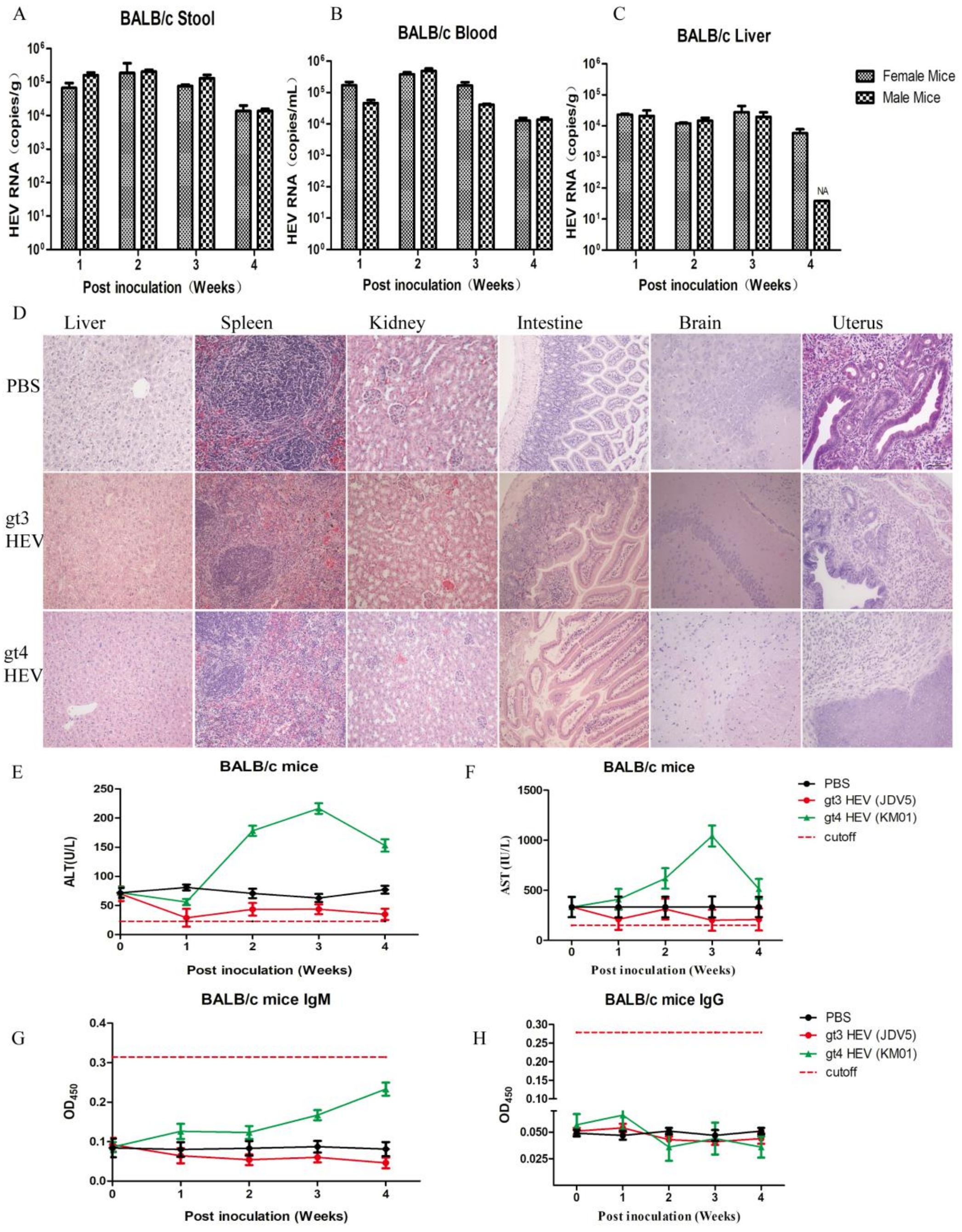

Supplementary Fig.3. Profile of HEV infection in C57BL/6 mice.

Splenomegaly was observed in C57BL/6 mice inoculated with gt4 HEV (A). Histopathological damage in C57BL/6 mice (H&E) (B). The activities of ALT (C) and AST (D) in C57BL/6 mice. The anti-HEV IgM (E) and IgG (F) antibodies in C57BL/6 mice inoculated with PBS, gt3 HEV or gt4 HEV.

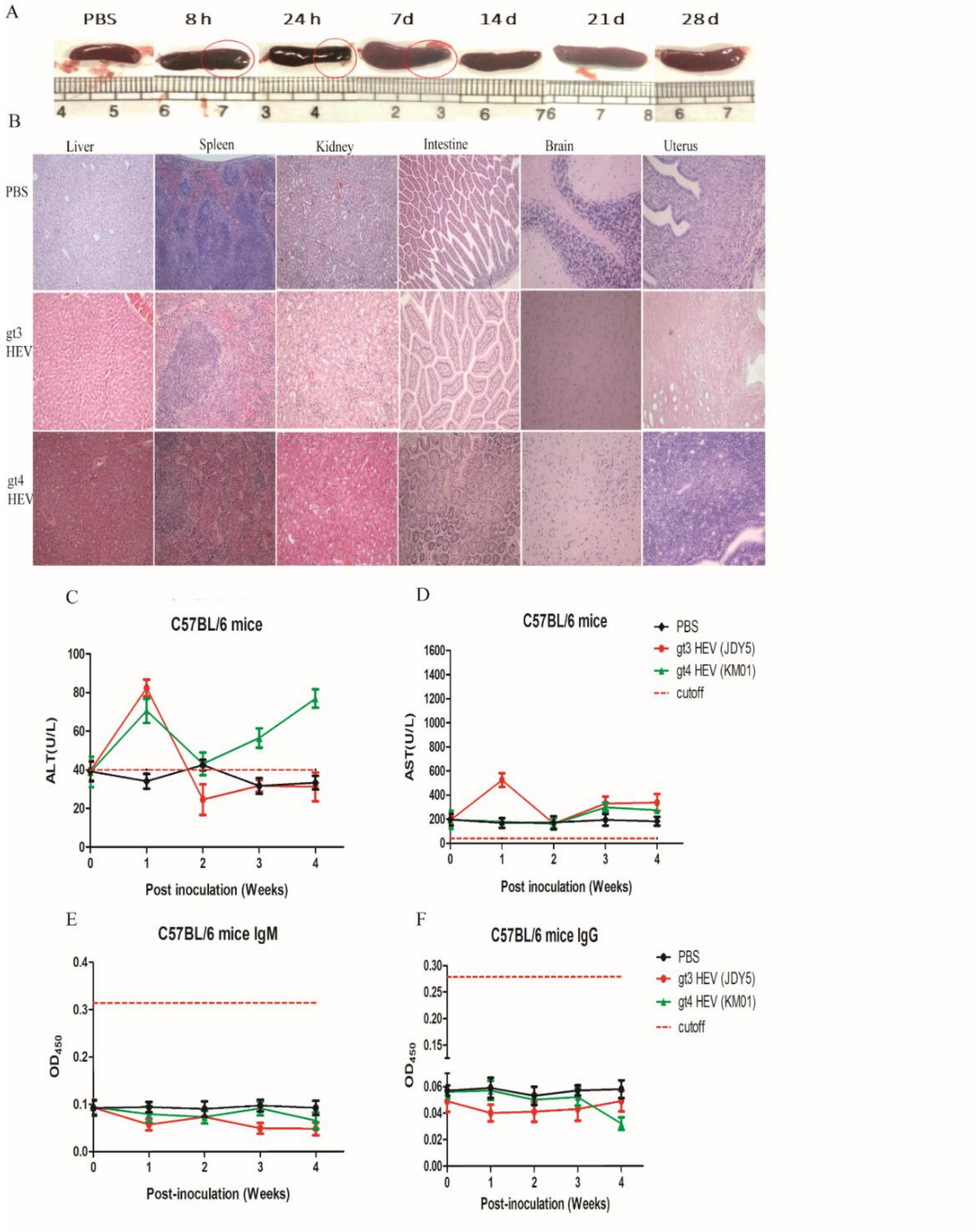

Supplementary Fig.4.Change in liver enzyme activities.

The activities of AST (A), ALT (B), AST/ALT (C), and T-BiL (D) in gt4 HEV-infected BALB/c mice treated with the indicated anti-HEV drugs.

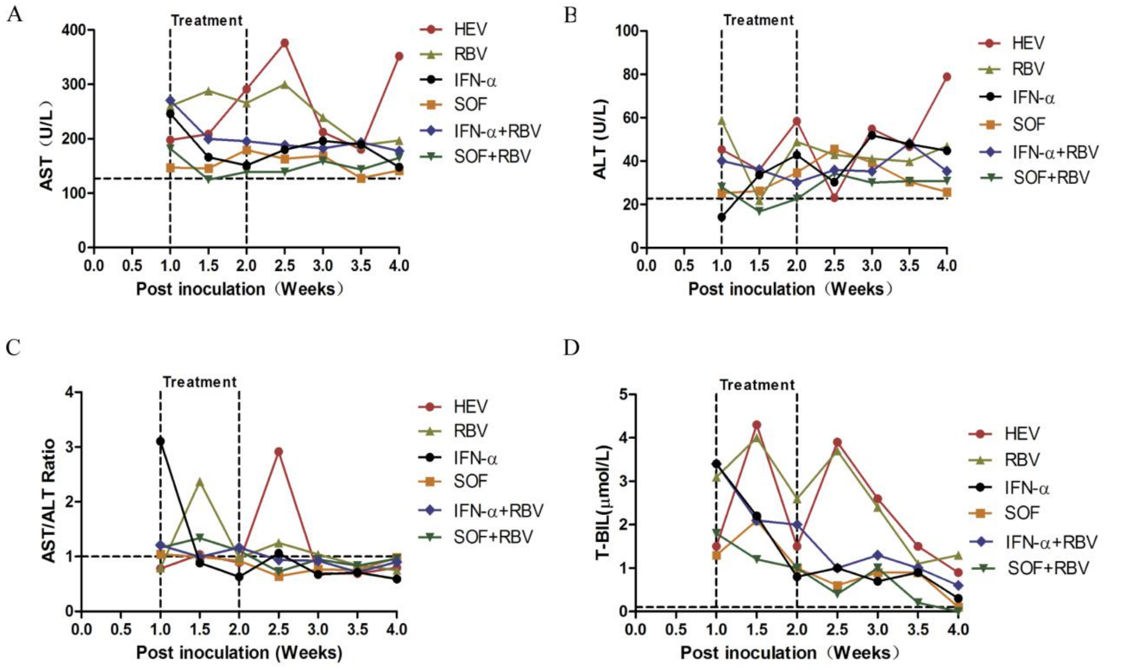

Supplementary Fig.5. Comparison of amino acids of HEV strains that result in effective infections with strains result in dull infection.

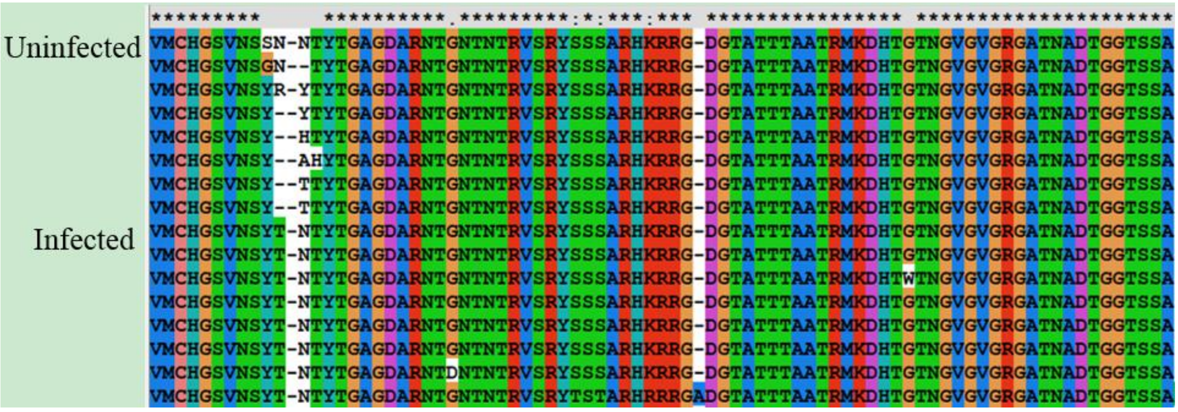

Supplement: Supplementary file 1 [file Data_Sheet_1.pdf]
